# Supplementary material for: Mining Heat-Resistant Key Genes of Peony Based on Weighted Gene Co-Expression Network Analysis
Source: Genes (Basel). 2024 Mar 21;15(3):383. doi: 10.3390/genes15030383 (PMC10970469; doi:10.3390/genes15030383)
Supplement: Supplementary file 1 [file genes-15-00383-s001.zip › genes-2856626-supplementary.pdf]

tatistical table of the number of up-regulated and down-regulated transcription factors in the comparative group at the same time

Table S1 Statistical table of the number of transcription factors up and down in the comparison group at the same time

**nparison**	**HS0h vs CK0h**			**HS2h vs CK2h**			**HS6h vs CK6h**			**HS12h vs CK12h**			**HS24h vs CK24h**		
's family	total	up	down	total	up	down	total	up	down	total	up	down	total	up	down
->AP2/ERF-AP2	-	-	-	2	-	2	-	-	-	-	-	-	-	-	-
->AP2/ERF-ERF	13	13	-	27	11	16	22	9	13	26	14	12	28	13	15
33->B3	3	3	-	9	-	9	7	-	7	8	-	8	7	2	5
->B3-ARF	-	-	-	1	-	1	1	-	1	-	-	-	1	-	1
BES1	-	-	-	-	-	-	-	-	-	-	-	-	1	1	-
bHLH	2	1	1	15	-	15	12	1	11	17	1	16	13	1	12
bZIP	2	-	2	6	2	4	6	1	5	8	-	8	7	1	6
->C2C2-Dof	2	-	2	6	1	5	5	1	4	6	-	6	8	1	7
C2C2-GATA	-	-	-	4	1	3	3	-	3	4	-	4	5	-	5
->C2C2-LSD	-	-	-	-	-	-	1	-	1	1	-	1	1	-	1
C2C2-YABBY	-	-	-	1	-	1	2	-	2	2	-	2	1	-	1
C2H2	8	-	8	18	3	15	17	1	16	22	2	20	14	2	12
C3H	-	-	-	3	1	2	4	1	3	8	1	7	6	1	5
CPP	-	-	-	2	1	1	1	1	-	2	1	1	-	-	-
DBB	-	-	-	1	1	-	-	-	-	1	1	-	-	-	-
CSD	-	-	-	-	-	-	1	1	-	-	-	-	-	-	-
DBP	-	-	-	2	1	1	2	1	1	2	1	1	2	1	1
E2F-DP	-	-	-	2	-	2	1	-	1	1	-	1	2	-	2
FAR1	1	1	-	5	4	1	4	4	-	9	8	1	12	12	-
GARP-G2-like	-	-	-	5	1	4	2	-	2	3	-	3	5	2	3
GeBP	-	-	-	-	-	-	-	-	-	2	1	1	1	-	1

Appendix S2 Statistical table of GO and KEGG enrichment related to stress response of each module

Table S2A GO functional enrichment related to stress response in each module

Module	GO ID	Description	GeneRatio	FDR
Black	GO:0009733	response to auxin	11/210	3.49E-09
	GO:0009735	response to cytokinin	9/210	4.83E-07
	GO:0009739	response to gibberellin	5/210	1.43E-05
	GO:0009751	response to salicylic acid	6/210	2.01E-05
	GO:0080167	response to karrikin	5/210	0.000138
	GO:0009737	response to abscisic acid	8/210	0.000196
	GO:0009611	response to wounding	6/210	0.000393
	GO:0033274	response to vitamin B2	2/210	0.000484
	GO:0009753	response to jasmonic acid	4/210	0.002034
	GO:0009723	response to ethylene	4/210	0.002040
Blue	GO:0009735	response to cytokinin	29/1391	8.25E-13
	GO:0009737	response to abscisic acid	23/1391	0.000228
	GO:0009733	response to auxin	13/1391	0.015474
	GO:0043247	telomere maintenance in response to DNA damage	2/1391	0.021724
Brown	GO:0009737	response to abscisic acid	57/1564	1.62E-26
	GO:0006952	defense response	65/1564	8.71E-25
	GO:0080167	response to karrikin	28/1564	7.55E-20
	GO:0009611	response to wounding	33/1564	1.09E-15
	GO:0009735	response to cytokinin	30/1564	8.39E-13
	GO:0009753	response to jasmonic acid	15/1564	1.77E-06
	GO:0009751	response to salicylic acid	15/1564	3.32E-06
	GO:0009723	response to ethylene	14/1564	9.66E-06
	GO:0009741	response to brassinosteroid	8/1564	1.23E-05
	GO:0071215	cellular response to abscisic acid stimulus	8/1564	8.86E-05
**Module**	**GO ID**	**Description**	**GeneRatio**	**FDR**

Cyan	GO:0009737	response to abscisic acid	4/64	0.006219
	GO:0080167	response to karrikin	2/64	0.013056
	GO:0042770	signal transduction in response to DNA damage	1/64	0.022230
	**GO:0034605**	**cellular response to heat**	**2/64**	**0.030290**
Green	GO:0009735	response to cytokinin	11/328	1.20E-06
	GO:1902884	positive regulation of response to oxidative stress	2/328	0.008439
	GO:0051788	response to misfolded protein	2/328	0.034165
	GO:0071244	cellular response to carbon dioxide	1/328	0.034165
Greenyellow	GO:0006952	defense response	3/59	0.023757
	GO:0010468	regulation of gene expression	2/59	0.026097
Grey60	GO:0009751	response to salicylic acid	1/24	1.91E-03
	GO:0010037	response to carbon dioxide	1/24	3.40E-03
	GO:0006952	defense response	1/24	1.08E-02
	GO:0009753	response to jasmonic acid	1/24	2.12E-02
	GO:0009723	response to ethylene	1/24	2.12E-02
Lightgreen	**GO:0009408**	**response to heat**	**1/15**	**0.019175**
Lightyellow	GO:0034599	cellular response to oxidative stress	6/26	4.71E-08
	GO:0006979	response to oxidative stress	6/26	5.64E-08
	GO:0009753	response to jasmonic acid	6/26	0.017705
	GO:0009723	response to ethylene	6/26	0.017705
	GO:0009751	response to salicylic acid	6/26	0.018312
	GO:0009611	response to wounding	6/26	0.028472
Magenta	GO:0009737	response to abscisic acid	4/124	0.021673
	GO:0009735	response to cytokinin	3/124	0.028075
	GO:0009410	response to xenobiotic stimulus	1/124	0.040144
**Module**	**GO ID**	**Description**	**GeneRatio**	**FDR**

Magenta	GO:0071369	cellular response to ethylene stimulus	1/124	0.045417
Midnightblue	GO:0006970	response to osmotic stress	3/36	0.001015
	GO:0070413	trehalose metabolism in response to stress	2/36	0.001015
	GO:0006952	defense response	4/36	0.001421
	GO:0009611	response to wounding	3/36	0.001421
	GO:0009751	response to salicylic acid	2/36	0.006295
	GO:0010037	response to carbon dioxide	1/36	0.007911
	GO:0047484	regulation of response to osmotic stress	1/36	0.014638
	GO:0031347	regulation of defense response	1/36	0.017353
	GO:0009741	response to brassinosteroid	1/36	0.017361
	GO:0009737	response to abscisic acid	2/36	0.017668
Pink	GO:0006979	response to oxidative stress	3/61	0.023136
Purple	GO:0009723	response to ethylene	3/105	0.004724
	GO:0070413	trehalose metabolism in response to stress	2/105	0.004780
	GO:0009741	response to brassinosteroid	2/105	0.006242
Red	GO:0009737	response to abscisic acid	13/371	7.59E-06
	GO:0009753	response to jasmonic acid	8/371	7.59E-06
	GO:0009751	response to salicylic acid	8/371	1.02E-05
	GO:0080167	response to karrikin	6/371	0.000327
	GO:0009723	response to ethylene	5/371	0.003233
	GO:0006979	response to oxidative stress	9/371	0.010205
	GO:0009739	response to gibberellin	3/371	0.018647
Salmon	GO:0009751	response to salicylic acid	4/58	4.85E-05
**Module**	**GO ID**	**Description**	**GeneRatio**	**FDR**

Salmon	GO:0009733	response to auxin	3/58	0.004336
	GO:0080167	response to karrikin	2/58	0.010037
	GO:0009753	response to jasmonic acid	2/58	0.010773
	GO:0009723	response to ethylene	2/58	0.010773
	GO:0009611	response to wounding	2/58	0.022957
Tan	GO:0009735	response to cytokinin	4/52	0.001980
	GO:0009739	response to gibberellin	2/52	0.008496
	GO:0009744	response to sucrose	2/52	0.008496
	**GO:0006950**	**response to stress**	**2/52**	**0.024999**
	GO:0000749	response to pheromone triggering conjugation with cellular fusion	1/52	0.035051
	GO:0009737	response to abscisic acid	2/52	0.036350
	**GO:0009408**	**response to heat**	**2/52**	**0.042258**
Turquoise	GO:0009735	response to cytokinin	45/2721	1.30E-16
	GO:0080167	response to karrikin	29/2721	2.09E-14
	**GO:0006950**	**response to stress**	**41/2721**	**1.38E-13**
	GO:0009611	response to wounding	40/2721	1.44E-13
	GO:0009737	response to abscisic acid	51/2721	1.09E-11
	GO:0009753	response to jasmonic acid	27/2721	1.18E-11
	GO:0009744	response to sucrose	16/2721	6.28E-07
	GO:0009739	response to gibberellin	12/2721	0.000180
	GO:0009733	response to auxin	24/2721	0.000462
	GO:0071446	cellular response to salicylic acid stimulus	5/2721	0.000779
Yellow	**GO:0009408**	**response to heat**	**114/1149**	**8.26E-88**
	GO:0042542	response to hydrogen peroxide	54/1149	5.19E-46
	**GO:0006950**	**response to stress**	**46/1149**	**2.60E-32**
	GO:0009266	response to temperature stimulus	17/1149	2.29E-18
**Module**	**GO ID**	**Description**	**GeneRatio**	**FDR**

Yellow	GO:0009737	response to abscisic acid	27/1149	3.77E-08
	GO:0000302	response to reactive oxygen species	10/1149	3.20E-05
	GO:0009739	response to gibberellin	8/1149	0.000270
	GO:0009723	response to ethylene	10/1149	0.000582
	GO:0080167	response to karrikin	9/1149	0.001346
	GO:0009611	response to wounding	13/1149	0.001687

Table S2B KEGG functional enrichment related to stress response in each module

Module	KEGG ID	Description	GeneRatio	FDR
Black	02010	ABC transporters	8/70	1.87E-07
	00062	Fatty acid elongation	6/70	2.63E-07
	00592	alpha-Linolenic acid metabolism	4/70	0.000833
	00982	Drug metabolism - cytochrome P450	4/70	0.003656
	00980	Metabolism of xenobiotics by cytochrome P450	4/70	0.004368
	00983	Drug metabolism - other enzymes	4/70	0.014158
	00906	Carotenoid biosynthesis	2/70	0.014608
	00944	Flavone and flavonol biosynthesis	1/70	0.016054
	00480	Glutathione metabolism	4/70	0.016054
Blue	99976	Replication and repair	40/534	4.08E-27
	00195	Photosynthesis	17/534	7.73E-11
	00710	Carbon fixation in photosynthetic organisms	26/534	3.62E-07
	00540	Lipopolysaccharide biosynthesis	5/534	0.000151
	00680	Methane metabolism	19/534	0.000315
	00196	Photosynthesis - antenna proteins	7/534	0.002594
	00630	Glyoxylate and dicarboxylate metabolism	23/534	0.004000
Brown	00196	Photosynthesis - antenna proteins	76/678	2.4e-111
	00195	Photosynthesis	22/678	1.99e-14
	04075	Plant hormone signal transduction	22/678	3.57e-12
	00906	Carotenoid biosynthesis	11/678	6.11e-08
	00902	Monoterpenoid biosynthesis	8/678	4.43e-06
	00901	Indole alkaloid biosynthesis	6/678	1.12e-05
	00460	Cyanoamino acid metabolism	14/678	1.61e-05

Module	KEGG ID	Description	GeneRatio	FDR
Brown	00940	Phenylpropanoid biosynthesis	16/678	1.75e-05
	00053	Ascorbate and aldarate metabolism	11/678	2.04e-05
	00943	Isoflavonoid biosynthesis	4/678	0.000247
green	00195	Photosynthesis	10/171	1.86e-08
	00630	Glyoxylate and dicarboxylate metabolism	11/171	0.013113
	00073	Cutin, suberine and wax biosynthesis	3/171	0.027191
	04137	Mitophagy - animal	5/171	0.044994
	99985	Amino acid metabolism	2/171	0.044994
greenyellow	00195	Photosynthesis	3/20	0.008439
grey60	00943	Isoflavonoid biosynthesis	1/7	0.034165
	04075	Plant hormone signal transduction	1/7	0.034165
	00900	Terpenoid backbone biosynthesis	1/7	0.023757
	00270	Cysteine and methionine metabolism	1/7	0.026097
lightcyan	00195	Photosynthesis	2/4	1.91E-03
lightcyan	00710	Carbon fixation in photosynthetic organisms	1/4	3.40E-03
	00630	Glyoxylate and dicarboxylate metabolism	1/4	1.08E-02
lightyellow	00196	Photosynthesis - antenna proteins	4/9	3.28e-09
	00900	Terpenoid backbone biosynthesis	2/9	0.000998
	01051	Biosynthesis of ansamycins	1/9	0.008786
	00195	Photosynthesis	1/9	0.017292
	04142	Lysosome	1/9	0.042798
	00030	Pentose phosphate pathway	1/9	0.042798
	00710	Carbon fixation in photosynthetic organisms	1/9	0.042798

Module	KEGG ID	Description	GeneRatio	FDR
pink	00196	Photosynthesis - antenna proteins	3/32	0.000935
	04064	NF-kappa B signaling pathway	2/32	0.033423
	00630	Glyoxylate and dicarboxylate metabolism	4/32	0.033423
red	00521	Streptomycin biosynthesis	7/128	1.13e-05
	00902	Monoterpenoid biosynthesis	4/128	0.000235
	00562	Inositol phosphate metabolism	7/128	0.000235
	00945	Stilbenoid, diarylheptanoid and gingerol biosynthesis	2/128	0.005551
	00940	Phenylpropanoid biosynthesis	5/128	0.007361
	00909	Sesquiterpenoid and triterpenoid biosynthesis	3/128	0.009366
	00941	Flavonoid biosynthesis	2/128	0.022217
	00410	beta-Alanine metabolism	5/128	0.027393
	00053	Ascorbate and aldarate metabolism	3/128	0.029614
	00981	Insect hormone biosynthesis	2/128	0.029631
tan	00480	Glutathione metabolism	3/26	0.042637
	04075	Plant hormone signal transduction	2/26	0.042637
	04016	MAPK signaling pathway - plant	2/26	0.042637
	00982	Drug metabolism - cytochrome P450	2/26	0.042637
	00980	Metabolism of xenobiotics by cytochrome P450	2/26	0.042637
	99976	Replication and repair	2/26	0.042637
turquoise	00195	Photosynthesis	42/1347	4.68e-27
	00902	Monoterpenoid biosynthesis	17/1347	2.94e-13

Module	KEGG ID	Description	GeneRatio	FDR
turquoise	00730	Thiamine metabolism	29/1347	5.48e-12
	00710	Carbon fixation in photosynthetic organisms	54/1347	9.36e-12
	00630	Glyoxylate and dicarboxylate metabolism	66/1347	7.33e-11
	00460	Cyanoamino acid metabolism	26/1347	2.33e-09
	00053	Ascorbate and aldarate metabolism	17/1347	1.73e-06
	00940	Phenylpropanoid biosynthesis	25/1347	2.60e-06
	00592	alpha-Linolenic acid metabolism	19/1347	1.13e-05
	04075	Plant hormone signal transduction	20/1347	1.20e-05
yellow	04010	MAPK signaling pathway	56/557	1.08e-31
	04144	Endocytosis	58/557	6.95e-20
	04151	PI3K-Akt signaling pathway	41/557	2.81e-16
	04217	Necroptosis	34/557	6.32e-14
	00982	Drug metabolism - cytochrome P450	16/557	3.11e-06
	00980	Metabolism of xenobiotics by cytochrome P450	16/557	8.30e-06
	04075	Plant hormone signal transduction	13/557	8.30e-06
	00983	Drug metabolism - other enzymes	16/557	0.000590
	00940	Phenylpropanoid biosynthesis	12/557	0.000729
	03060	Protein export	11/557	0.001493

Appendix S3 Color meaning table of 19 modules

Table S3 Color meaning table of 19 modules

Number	Color	Module name
1		Blue module
2		Tan module
3		Salmon module
4		Yellow module
5		Cyan module
6		Black module
7		Purple module
8		Lightcyan module
9		Lightyellow module
10		Greenyellow module
11		Magenta module
12		Midnightblue module
13		Grey60 module
14		Lightgreen module
15		Turquoise module
16		Pink module
17		Red module
18		Brown module

19		Green module
